# Revealing Three Stages of DNA-Cisplatin Reaction by a Solid-State Nanopore

**DOI:** 10.1038/srep11868

**Published:** 2015-07-07

**Authors:** Zhi Zhou, Ying Hu, Xinyan Shan, Wei Li, Xuedong Bai, Pengye Wang, Xinghua Lu

**Affiliations:** 1 Beijing National Laboratory for Condensed-Matter Physics and Institute of Physics, Chinese Academy of Sciences, Beijing 100190, People’s Republic of China; 2 Collaborative Innovation Center of Quantum Matter, Beijing 100190, People’s Republic of China

## Abstract

The dynamic structural behavior in DNA due to interaction with cisplatin is essential for the functionality of platinum-based anti-cancer drugs. Here we report a novel method to monitor the interaction progress in DNA-cisplatin reaction in real time with a solid-state nanopore. The interaction processes are found to be well elucidated by the evolution of the capture rate of DNA-cisplatin complex, which is defined as the number of their translocation events through the nanopore in unit time. In the first stage, the capture rate decreases rapidly due to DNA discharging as the positive-charged hydrated cisplatin molecules initially bond to the negative-charged DNA and form mono-adducts. In the second stage, by forming di-adducts, the capture rate increases as DNA molecules are softened, appears as the reduced persistence length of the DNA-cisplatin adducts. In the third stage, the capture rate decreases again as a result of DNA aggregation. Our study demonstrates a new single-molecule tool in exploring dynamic behaviors during drug-DNA reactions and may have future application in fast drug screening.

Platinum-based drugs are widely used in nowadays cancer chemotherapy^1^,^2^,^3^. It has been revealed that the drug molecules disable the physiologic activities of DNA and induce cell apoptosis by bonding with the N7 atom of guanine or adenine along a DNA molecule to form mono- and di-adducts^4^,^5^,^6^. Certain cancers, such as testicular cancer, can be cured with great success by cisplatin, one of the most effective platinum-based drugs^7^,^8^,^9^,^10^. Applying similar drugs to cure other cancers is believed to be of extraordinary potential^7^. To design new platinum-based drugs, the underlying mechanism governing dynamic DNA-drug interaction has to be fully understood^11^,^12^. Significant progress has been made in the past years in probing such interaction at the microscopic scale^13^,^14^,^15^,^16^,^17^,^18^. The kinetics of DNA-cisplatin interaction has been revealed by measuring the characteristic nuclear magnetic resonance (NMR) signals of DNA-cisplatin mono- and di-adducts along the reaction period^13^. Single-molecule stretching experiments with optical^14^ or magnetic tweezers^15^ have demonstrated that the persistence length of a DNA molecule is notably reduced due to the interaction with cisplatin molecules. DNA condensation under high cisplatin concentration has also been imaged by atomic force microscope (AFM) studies^15^,^16^. Nonetheless, real-time monitoring of DNA structural behavior during reaction with cisplatin has not been easy with all these known methods, and new tools to trace such reaction process are strongly desired.

Nanopore is a novel technology that detects a single biomolecule by monitoring conductance blockade due to translocation of the molecule through a nanometer-sized pore^19^. Such devices have been employed to explore various biomolecules such as DNA^20^, RNA^21^, protein^22^ and their complexes^23^,^24^, especially DNA sequencing with MspA nanopores^25^,^26^. It is also readily applicable for the study of molecule-molecule interactions and their dynamics^27^. In this study, we employ solid-state nanopores to probe the dynamic progress that DNA interacts with cisplatin molecules. The advantage of solid-state nanopore lies in its size control ability and long-term stability. The DNA-cisplatin adducts translocate through the nanopore and the capture rate of adducts is monitored continuously for a day or two. The temporal evolution in the capture rate illuminates three stages of DNA-cisplatin interaction. The evolutions of charge, persistence length, and effective diameter of DNA molecules in respective stages are quantitatively revealed with the aid of a unified physical model.

## Physical model

**Figure 1:**
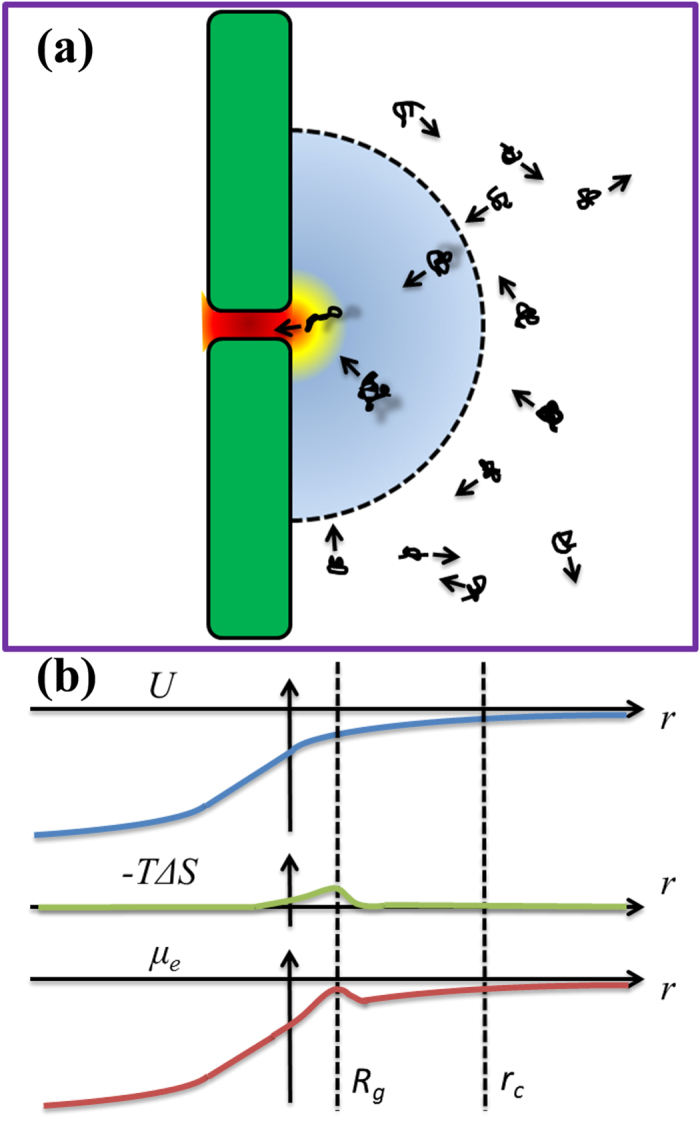
(**a**) Schematic for the capture of DNA molecules into the nanopore. The dash line illustrates the absorbing boundary within which the driving force prevails over the diffusion effect. The arrows illustrate the motion of DNA molecules. The entropy barrier is near the pore opening by a distance of *R*_*g*_. (**b**) The distribution of electric potential (*U*), entropic cost (*−TΔS*), and electrochemical potential (*μ*_*e*_) as a function of distance *r* from the pore center. The dash lines indicate the position of entropy barrier and absorbing boundary.

As described by Wanunu *et al.*^28^, the transport path of DNA through a nanopore can be divided into five steps: (i) free diffusion to a semi-spherical absorbing boundary, (ii) biased diffusion to the nanopore, (iii) DNA threading into the nanopore, (iv) DNA translocation through the nanopore, and (v) DNA escaping away from the nanopore. The translocation throughput is determined by the absorbing boundary and the threading entropic barrier. Figure 1(a) illustrates both critical factors in capturing a DNA molecule into a nanopore. The entropy barrier is located near the pore opening by a distance on the order of *R*_*g*_, the gyration radius of the DNA molecule. The distribution of electric potential, entropic cost, and electrochemical potential as a function of distance from the pore opening are shown in Fig. 1(b). Quantitatively, the number of translocation events per unit time, or the capture rate *J*, can be described by Kramers’ theory as
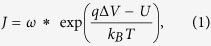
where *q* is the effect charge of DNA end segment, Δ*V* is the applied voltage across the electrodes, *U* is the entropic barrier energy, *k*_*B*_ is Boltzmann constant, and *T* is the absolute temperature. The collision frequency *ω* is proportional to the throughput from the absorbing boundary and can be written as *πcμd*^2^Δ*V*/4*h*^28^, where *c* is DNA concentration, *μ* is electrophoretic mobility, *d* and *h* are diameter and thickness of the nanopore. The electrophoretic mobility *μ* is proportional to 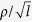, where *ρ* and *l* are linear charge density and persistence length of DNA molecules. Due to steric hindrance effect, the success rate of threading a DNA end segment into the pore is better described as proportional to (*d*−2*r*)^2^, instead of *d*^2^, where *r* is the effective radius of the DNA molecule. Combining the pre-exponential invariant as a constant *A*, the total capture rate *J* is derived as follows:
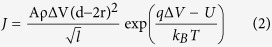


## Experiments and Results

Our experiments are carried out with 10 *kbp* double-stranded DNA (Purchased from Thermo Scientific, catalog number: SM1751). In cell, cisplatin (*cis*-[Pt(NH_3_)_2_(Cl)_2_]) is activated by taking off the chloride ligands to form *cis*-[Pt(NH_3_)_2_(H_2_O)_2_] or *cis*-[Pt(NH_3_)_2_(H_2_O)Cl], before interacting with DNA^7^. To be consistent with such procedure, the cisplatin used in our experiments is pre-activated as well with the method described by Hou *et al.*^15^. It is then diluted in sodium acetate buffer (1 *M* NaAc, 10 *mM* HEPES and *pH* = 7.8) which contains 1 *nM* 10 *kbp* double strand DNA (dsDNA), corresponding to a 10 *μM* basepair concentration *C*_*bp*_. The sodium acetate is chosen to replace the chloride as used in normal nanopore experiments. The relative concentration of diluted cisplatin, *α*, is defined as the ratio of the absolute concentration of cisplatin to the basepair concentration *C*_*bp*_. Nanopores with diameter between 5 to15 *nm* are fabricated on 50 *nm* thick silicon nitride membranes and show good stability for long time recording (within 20% conductance variation) in the NaAc-HEPES buffer (see supplementary information SI-1). A pair of silver acetate electrodes with applied voltage are used to drive the electrophoresis of DNA-cisplatin adducts. Translocation of DNA-cisplatin adducts with different conformations are then readily detected by monitoring the ionic current (see supplementary information SI-2).

**Figure 2:**
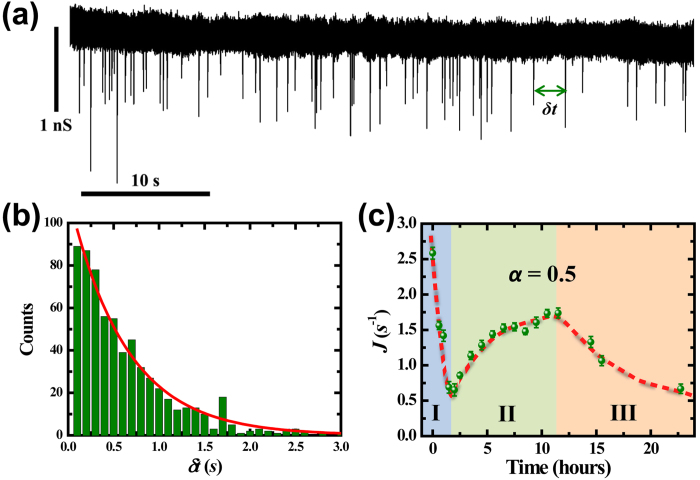
(**a**) Typical current trace with translocation events of DNA-cisplatin adducts through a SiN nanopore. The pore diameter is 5.8 *nm* and the driving voltage is 500 *mV*. (**b**) Histogram distribution of time interval *δt* between adjacent events. The red curve is the exponential fit with capture rate of 1.59 ± 0.07 *s*^*−1*^. (**c**) Evolution of capture rate as a function of reaction time. The red curve and the colored background are guided for the eye.

Figure 2a presents a typical current trace with translocation events of DNA-cisplatin adducts, in which the relative ratio *α* equals 0.5. The translocation events are represented by the spikes in the trace. The time interval between adjacent events, *δt*, is statistically analyzed with the histogram plot, as shown in Fig. 2b. The distribution can be fitted with an exponential function, *P*(*δt*) = *N* * exp(−*J* * *δt*), where *N* is a normalization constant and *J* is the capture rate which is of our main interest^29^. Fitting the data in Fig. 2b derives a capture rate of 1.59 ± 0.07 *s*^*−1*^ (events per second). To investigate the dynamic progress of DNA-cisplatin interaction, the temporal variation in capture rate is measured as a function of reaction time. Figure 2c shows the evolution of the capture rate along a period of 25 hours for a 5.8 *nm* nanopore. Three stages are clearly illustrated. The capture rate reduces rapidly in the first a few hours (stage I), it then increases to a saturated value in the following 10 hours or so (stage II), and decreases again (stage III). Such feature is observed for all experiments with *α* varies from 0.5 up to 10. The characteristic feature in capture rate reflects different dynamic behaviors in each stage: DNA discharging, DNA softening, and DNA aggregation, as we explain in details in the following sections.

### Stage I: DNA discharging

**Figure 3:**
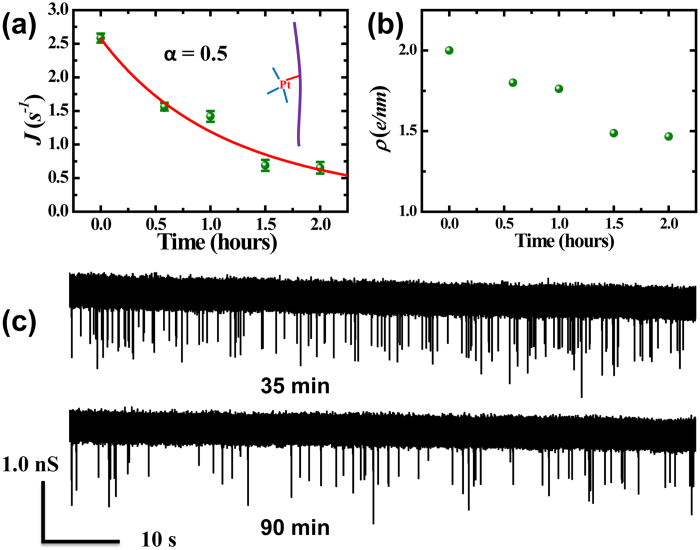
Stage I. (**a**) Temporal evolution of capture rate in stage I. The data is fitted with a second-order reaction model (red line, see SI-3 for the details). Inset: schematic of DNA-cisplatin mono-adducts. (**b**) The evolution of the derived linear charge density *ρ(t)* in the first stage. (**c**) Current traces taken at 35 and 90 minutes since injection of cisplatin molecules.

DNA molecules are negatively charged with about 2 electrons per *nm* in the sodium acetate buffer, similar to that in 1 *M* KCl electrolyte environment^30^. When cisplatin is mixed with DNA solution, they diffusively approach the DNA molecules, firstly bond to the guanine base to form mono-adducts by which only one covalent bond is established with each attached cisplatin molecule (see the schematic inset of Fig. 3a). Since each cisplatin molecule (hydrated form) carries two positive charges, the effective charge density of DNA-cisplatin adducts reduces as more and more cisplatin molecules are attached. Consider charge as the distinct variable in stage I, the capture rate *J(t)* in equation (2) can be simplified to:
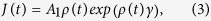
where *A*_*1*_ is a constant, *ρ(t)* is the effect charge density of DNA-cisplatin mono-adducts, and 
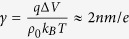
 if Δ*V* equals 500 *mV*, as derived from data in reference^28^. Since the formation of mono-adduct is a second order reaction, the explicit form of *ρ(t),* and then *J(t),* can be derived (see SI-3 for the detailed derivation). Figure 3a shows the zoom-in plot of stage I in Fig. 2c. Fitting the data derives a reaction rate of 0.22 ± 0.08 *μM*^−1^ *h*^−1^ for the initial bonding of cisplatin to DNA (see SI-3 for details). The typical time of this period is about 70 minutes. Figure 3(b) presents the change in charge density during the stage. Figure 3(c) shows two current traces taken at 35 and 90 minutes since injection of cisplatin, where the reduction in capture rate can be clearly seen.

We note that the electrostatic interaction with charged molecules may change the persistence length of DNA, which has been assumed to be a constant in this stage. Measurements with magnetic tweezers indicate that the persistence length reduces monotonically during the first few hours^15^. However, according to equation (2), the reduction in persistence length tends to increase the capture rate, which is opposite to our observation. Thus, the change in electrostatic persistence length has negligible effect on the characteristic features in this stage, i.e. discharging and decreasing in capture rate.

### Stage II: DNA softening

**Figure 4:**
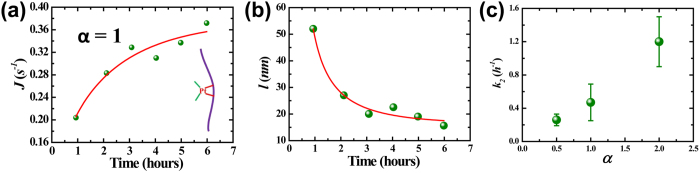
Stage II. (**a**) Capture rate versus reaction time. The driving voltage is 200 *mV* and the nanopore diameter is 5.3 *nm*. The solid line is the fitting result. Inset: schematic of DNA-cisplatin di-adducts. (**b**) Plot of the measured reduced persistence length versus reaction time. The solid line is the fitting result with equation (5). (**c**) Plot of the rate constant *k*_*2*_ versus concentration ratio *α*.

The bonded cisplatin molecule may have its second arm bond to a neighboring guanine or adenine base, forming a di-adduct (see the schematic image in the inset of Fig. 4a)^4^. The di-adduct bends the DNA molecule and decreases its persistence length. The linear charge density keeps constant during this stage, and the capture rate *J* can be simply represented as a function of persistence length:
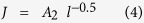
where *A*_*2*_ is a constant in the stage. The capture rate increases due to the reduction of persistence length. Figure 4a shows typical evolution of capture rate *J* in this stage. The concentration ratio *α* equals 1 in this data set and results are similar for *α* of 0.5 and 2.

The persistent length can be derived from equation (4) as 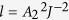. Figure 4b plots the converted persistence length *l* versus reaction time *t* in the second stage. The constant *A*_*2*_ is chosen such that *l* equals to that of a natural DNA in similar electrolyte condition, typically 52 *nm*, at the beginning of the stage. The persistence length *l*(*t*) depends on the concentration of the di-adducts and has the form of 
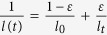
14,31,32, where ε = *C*_*t*_/*C*_2_, *l*_*0*_ and *l*_*t*_ are persistence lengths at the beginning and end of this stage, *C*_*t*_ is the di-adducts concentration at time *t*, and *C*_*2*_ is the final di-adducts concentration. If we assume that the di-adducts are generated following a simple exponential law with a rate constant *k*_*2*_, C_*t*_ = *C*_2_(1−*e*^−*k*^_2_^*t*^), the persistence length *l*(*t*) then have the following form:
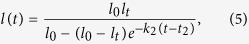
where *t*_2_ represents the beginning of stage II. Fitting data in Fig. 4b reveals a rate constant *k*_*2*_ of 0.47 *h*^*−1*^, *l*_*t*_ of 16.4 *nm*, and *t*_*2*_ of 0.93 *h*, which are in good agreement with previous results measured by single molecule stretching experiments^15^. The red curve in Fig. 4b is the fitting curve. It is interesting to observe that the rate constant *k*_*2*_ has a strong dependence on the concentration ratio *α*. Its value increases from 0.26 *h*^*−1*^ to 1.2 *h*^*−1*^ as *α* is increased from 0.5 to 2.0, as shown in Fig. 4c.

### Stage III: DNA aggregation

**Figure 5:**
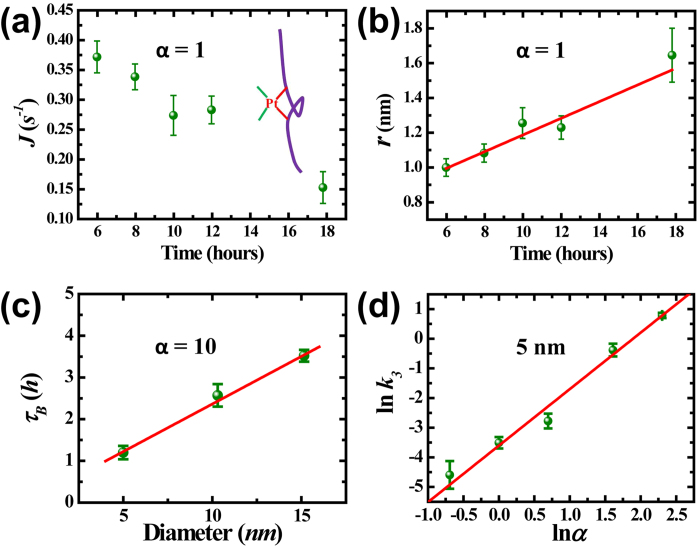
Stage III. (**a**) Capture rate *J* versus reaction time. Inset: schematic of DNA micro-loop and crosslink formed by di-adduct. *ΔV* = 200 *mV*, *d* = 5.3 *nm*. (**b**) Effect radius *r* versus reaction time. (**c**) Aggregation time *τ*_*B*_ versus nanopore diameter with concentration *α* = 10, Δ*V* = 500 *mV*. The solid lines in (**b**) and (**c**) are linear fits. (**d**) The correlation between rate constant *k*_*3*_ and concentration ratio *α*. Solid line is a linear fit with slope equals 1.9 ± 0.1.

Along a DNA molecule chain, some mono-adducts have no guanine or adenine bases as their neighbors and thus cannot form di-adducts in the second stage. However, they may bond to guanine or adenine base further away along the DNA molecular chain due to thermal fluctuation. By forming di-adducts with a further-away base, the drug molecules induces micro-loop structures and crosslinks (see the schematic inset of Fig. 5a), which eventually drive the DNA molecule to condense to a compact globule^15^. In such processes, the effective diameter of the DNA molecule increases, and then the free energy barrier of DNA being threaded into the solid-state nanopore increases as well. The steric hindrance effect plays a dominate role in determining the capture rate in this stage,
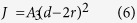
where *A*_*3*_ is a constant in this stage. The capture rate *J* decreases as the effective diameter 2*r* increases up to the nanopore diameter *d* by when DNA molecule will not translocate through the nanopore anymore. Figure 5a shows the evolution of capture rate in this stage for DNA sample mixed with 10 *μM* cisplatin (*α* = 1). The diameter of the pore is 5.3 *nm* and the driving voltage is 200 *mV*. The capture rate decreases from 22.5 minute^−1^ to 9 minute^−1^ in a period of 12 hours.

By setting the effective radius *r* to that of a natural DNA (1.0 *nm*) at the beginning of this stage, we derive the evolution of effective radius from capture rate in Fig. 5a. The results are shown in Fig. 5b, which suggests linear growth kinetics. A growth rate *k*_*3*_ of 0.05 ± 0.01 *nm/h* is revealed by linear fit of the data (solid line in Fig. 5b). Such linear growth feature can also be demonstrated by measuring the total aggregation time from beginning of incubation till the capture rate falls to zero, *τ*_*B*_, with nanopores of different diameters, as shown in Fig. 5c.The total aggregation time is linear with nanopore diameter, indicating a growth rate of 2.2 ± 0.2 *nm/h*. This significantly higher aggregation rate is due to the higher cisplatin concentration ratio (*α* = 10) as compared with that in Fig. 5a,b.

The sensitivity of aggregation process on the cisplatin concentration is further investigated. Figure 5d represents the correlation between the rate constant *k*_*3*_ and concentration ratio *α* in a log plot, for data taken in nanopores 5.3 ± 0.5 nm in diameter. The effect of natural degradation of DNA molecules has been corrected with a logistic regression model^33^ (see SI-4 for more details). Red line is a linear fit with a slope of 1.9 ± 0.1. This infers that the rate constant *k*_*3*_ is on the power law of *α* with an index of 1.9 ± 0.1. Recently, by imaging the DNA-cisplatin adducts with atomic force microscope, Hou *et al.*^15^ found that it took about 1 hour to form local links in DNA for high cisplatin concentration (*α* = 10, 770 *μM* cisplatin) and it took longer than 6 hours for low cisplatin concentration (*α* = 1, 77 *μM* cisplatin). The characteristic aggregation time in their study is consistent with our measurements by solid state nanopores.

## Discussion

Three stages of DNA-cisplatin interaction are revealed by monitoring the capture rate of DNA translocation through a solid-state nanopore. The reduction in capture rate in the first stage reveals a discharging process in DNA molecules due to the formation of mono-adducts. The molecular linear charge density is reduced by 25% for a concentration ratio of 0.5. In addition, the duration of DNA translocation events was enlarged in stage I (see supplementary information SI-5). The increase in capture rate in the second stage indicates the formation of di-adduct and the decrease in the persistence length of DNA molecules. The rate constant in forming di-adducts has a strong dependence on the concentration ratio. The aggregation of DNA molecules in the third stage increases its effective diameter and reduces the capture rate again. The current blockage amplitudes were increased due to cisplatin-DNA binding, but mostly during the first two stages (see supplementary information SI-6). There is no apparent increase in current blockage amplitude during stage III, probably because the loops formed in this stage are too loose to block the ionic current effectively.

Our study demonstrates solid-state nanopore as a new single-molecule tool in real-time monitoring of the dynamic structure changes in DNA molecules interacting with drug molecules. Further implementation of such method may help in not only revealing fundamental mechanism for DNA-molecule interaction, but also new drug design and pharmacokinetics.

## Methods

### Silver acetate electrodes

The silver acetate electrodes were prepared by immersing polished silver filaments (1 *mm* diameter) into saturated Fe(NO_3_)_3_ and NaAc solutions for 3 seconds alternatively for about 30 rounds. The electrodes were then immersed in 1 *M* NaAc solution for more than 12 hours to remove the residual oxide layer. When the as-fabricated electrodes were loaded into a home-built flow cell, the system was filled with 1 *M* NaAc electrolyte as soon as possible to avoid the electrodes being oxidized by air.

### Cisplatin activation

Cisplatin (Pt(NH_3_)_2_Cl_2_) was hydrated to form *cis*-Pt(NH_3_)_2_(H_2_O)_2_ by reacting with AgNO_3_ for 24 hours in the dark. Then, the AgCl precipitate was removed by centrifuging at 13000 *rpm* for 10 minutes by two times. The activated drug was kept under −15 *°C*. It was then diluted to the target concentration and incubated with DNA molecules during experiments.

### Experimental condition

The nanopores were fabricated in suspended silicon nitride membranes (50 *nm* thick, 50 *μm* x 50 *μm* in size) in a transmission electron microscope (JEM 2010F), supported by 500 *μm* thick silicon chips. Prior to be loaded into the flow cell, the fabricated nanopore chips were cleaned by piranha solution at 120 *°C* for about 30 minutes and then washed with de-ion water. After assembled the system and injected buffer, I-V curves and noise analysis were taken to test the performance of the nanopores. Only those nanopores with linear and symmetric I-V curves and stable base lines (low *1/f* noise) were selected for experiments.

When DNA-cisplatin mixtures were added in the chamber, a biased voltage (range from 200 to 500 *mV*) was applied across the electrodes and a current trace with blocked DNA translocation events was monitored. For each reaction time point, the current trace was recorded for a certain time (typically 10 minutes) and there were typically 1000 events for each reaction time, except in stage III, which is of low capture rate. During two reaction time points, the driving voltage is turned off and the time interval is typically more than 20 minutes. When the capture rate gets very low (or zero when the pore is blocked), the experiment was stopped.

The analog current signal was amplified by Axopatch 200B, filtered at 30 *kHz* and digitized at 250 *kHz* sampling rate by Axon 1440A. The DNA translocation events were extracted by a home-built Matlab program and analyzed with Origin 8.0.

## Additional Information

**How to cite this article**: Zhou, Z. *et al.* Revealing Three Stages of DNA-Cisplatin Reaction by a Solid-State Nanopore. *Sci. Rep.* **5**, 11868; doi: 10.1038/srep11868 (2015).

## Supplementary Material

Supplementary Information
