# Supplementary material for: Revealing Three Stages of DNA-Cisplatin Reaction by a Solid-State Nanopore
Source: Sci Rep. 2015 Jul 7;5:11868. doi: 10.1038/srep11868 (PMC4493569; doi:10.1038/srep11868)
Supplement: Supplementary Information [file srep11868-s1.pdf]

Supplementary information

Revealing Three Stages of DNA-Cisplatin Reaction by a Solid-State Nanopore

Zhi Zhou[1], Ying Hu[1], Xinyan Shan[1], Wei Li[1], Xuedong Bai[1,2], Pengye Wang[1], and Xinghua Lu[1,2]

1. *Beijing National Laboratory for Condensed-Matter Physics and Institute of Physics, Chinese Academy of Sciences, Beijing 100190, People's Republic of China*
2. *Collaborative Innovation Center of Quantum Matter, Beijing 100190, People's Republic of China*

E-mail: xhlu@iphy.ac.cn

SI-1 Stability of AgAc/Ag electrodes for nanopore experiment.

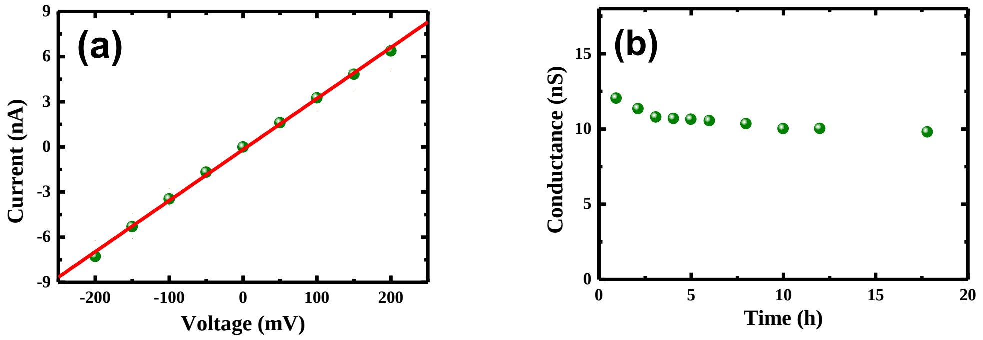

Figure SI-1 Stability of AgAc/Ag electrodes. (a) I-V curve of a 5 *nm* SiN nanopore in NaAc-HEPES buffer (1 *M* NaAc and 10 *mM* HEPES, *pH* = 7.8). Linear and symmetric feature is demonstrated by the linear fitting (red line).The fitted conductance is 34 *nS*. (b) Evolution in conductance for a 5 *nm* nanopore.

SI-2 Current trace of DNA-cisplatin adducts translocation through a solid-state nanopore.

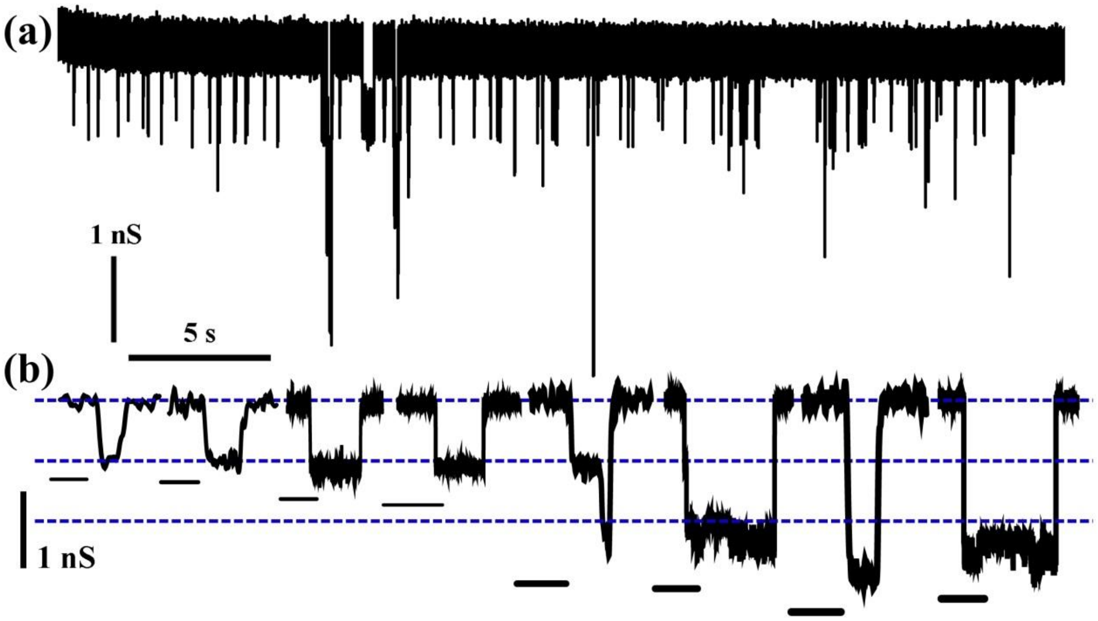

Figure SI-2 (a) Typical current trace of DNA-cisplatin adducts translocation through a SiN nanopore. The trace is recorded after 4-hour reaction. (b) Typical unfolded and folded translocation events. The time scale bars from left to right are 0.2, 0.4, 4, 40, 1, 40, 1, and 200 *ms*. ΔV = 500 *mV*, *d* = 5.6*nm*, and α = 2.

SI-3 Derivation of capture rate as a function of reaction time in stage I.

In stage I, the reaction between DNA and cisplatin is a second order reaction. We set the initial base-pair concentration as C_0, the active site concentration as C_a, the initial linear charge density as ρ_0, and the DNA-cisplatin mono-adducts concentrate as C_t which is a function of time t.

$$\begin{array}{lll} \text{cisplatin} + \text{DNA} & \xrightarrow{k_1} & \text{DNA-cisplatin} \\ \alpha C_0 \quad C_a & & 0 \\ \alpha C_0 - C_t \quad C_a - C_t & & C_t \end{array},$$

$$R = \frac{dC_t}{dt} = k_1 * (C_a - C_t) * (\alpha C_0 - C_t)$$

$$\Rightarrow C_t = \frac{\alpha C_0 C_a (1 - e^{k_1(\alpha C_0 - C_a)t})}{C_a - \alpha C_0 e^{k_1(\alpha C_0 - C_a)t}}$$

Then, the linear charge density of the DNA-cisplatin adducts $\rho(t)$ can be derived as:

$$\rho(t) = \frac{Q(t)}{L} = \frac{\rho_0 L - q_c * \frac{C_t}{C_0} * \frac{L}{b}}{L} = \rho_0 - \frac{q_c}{b} * \frac{C_t}{C_0};,$$

$$= \rho_0 - \frac{q_c}{b} * \frac{\alpha C_a (1 - e^{k_1(\alpha C_0 - C_a)t})}{C_a - \alpha C_0 e^{k_1(\alpha C_0 - C_a)t}}$$

Where b is the length of a DNA base-pair (0.34 *nm*), q_c is the effect charge of a cisplatin molecule (2*e*), and $\frac{C_t}{C_0} * \frac{L}{b}$ equals the number of cisplatin molecules bonded onto a single DNA molecule at time t. Therefore, the equation (2) in the main text can be written as:

$$J = A_1(\rho_0 - \frac{q_c}{b} * \frac{\alpha C_a (1 - e^{k_1(\alpha C_0 - C_a)t})}{C_a - \alpha C_0 e^{k_1(\alpha C_0 - C_a)t}}) \exp(\gamma * (\rho_0 - \frac{q_c}{b} * \frac{\alpha C_a (1 - e^{k_1(\alpha C_0 - C_a)t})}{C_a - \alpha C_0 e^{k_1(\alpha C_0 - C_a)t}})) \text{ (SI-1)},$$

where A_1 is a constant. Figure 3(a) was fitted with equation (SI-1), shown as the red curve. The fitted rate constant k_1 is about 0.22 ± 0.08 $\mu M^{-1} h^{-1}$. C_a is about 0.80 μM and final linear charge density $\rho_t = \rho_0 - \frac{q_c}{b} * \frac{C_a}{C_0} \approx 1.5\, e/nm$.

SI-4 Evolution of capture rate of untreated DNA molecules.

We consider the consumption of DNA molecules by bacterial. The growth of bacterial colony can be modeled by Logistic regression and has the formula as follows:

$$\frac{dN}{dt} = \lambda * N * \frac{K - N}{K}$$

Where λ defines growth rate of the bacterial colony and K represents environmental carrying capacity. By solving this equation and with $\mathrm{N_0 = N(t = 0)}$, we get

$$\mathrm{N}(t) = \frac{K}{1 + \frac{K - N_0}{N_0} e^{-\lambda t}}$$

The amount of DNA molecules in the sample volume is reduced as following

$$\frac{dC_d(t)}{dt} = -\beta \mathrm{N}(t) C_d(t) = \frac{-\beta K C_d(t)}{1 + \frac{K - N_0}{N_0} e^{-\lambda t}}$$

$$\frac{C_d(t)}{C_0} = \left(1 + \frac{N_0}{K}\left(e^{\lambda t} - 1\right)\right)^{-1}$$

The capture rate is proportional to the amount of DNA molecules, and the corrected function we used in the main text is as following:

$$\Gamma(t) = \frac{J(t)}{J_0} = \frac{C_d(t)}{C_0} = \frac{1+\chi}{1+\chi e^{\lambda t}} \quad \text{(SI-3)}$$

where $\chi = \frac{N_0}{K - N_0}$. Fitting of Figure SI-4 derives χ of 0.007 and λ of 0.24 h^{-1}.

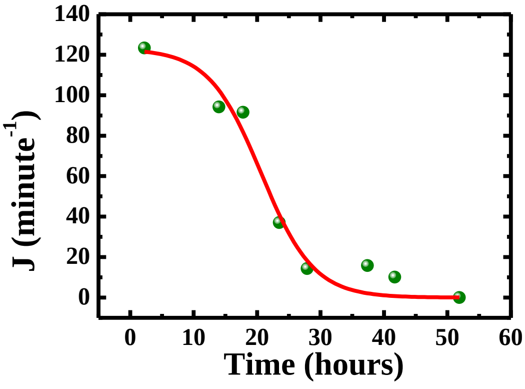

Figure SI-4 Evolution of capture rate of untreated DNA molecules as a function of time. The pore diameter is 5.6 *nm* and the driving voltage is 500 *mV*. The data is fitted with equation (SI-3) as shown as red solid line.

SI-5 Evolution of translocation duration in stage I.

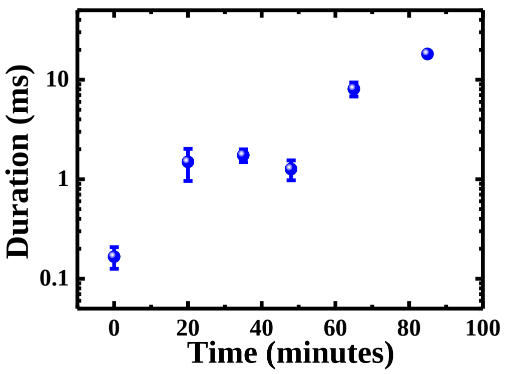

Figure SI-5 Plot of the average translocation duration versus reaction time in stage I. The pore diameter is 5 *nm* and the driving voltage is 500 *mV*, and $\alpha = 2$.

SI-6 Evolution of translocation current blockade.

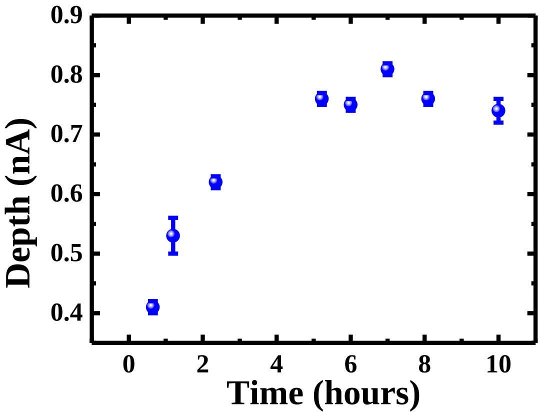

Figure SI-6 Plot of the average current blockade versus reaction time. The pore diameter is 5 *nm* and the driving voltage is 500 *mV*, and $\alpha = 2$. Stage III is from 5 to 10 hours.
